# Supplementary material for: PI16+ reticular cells in human palatine tonsils govern T cell activity in distinct subepithelial niches
Source: Nat Immunol. 2023 May 18;24(7):1138–48. doi: 10.1038/s41590-023-01502-4 (PMC10307632; doi:10.1038/s41590-023-01502-4)
Supplement: Supplementary file 1 — Reporting Summary [file 41590_2023_1502_MOESM1_ESM.pdf]

nature portfolio

Corresponding author(s): Burkhard Ludewig

Last updated by author(s): Mar 31, 2023

Reporting Summary

Nature Portfolio wishes to improve the reproducibility of the work that we publish. This form provides structure for consistency and transparency in reporting. For further information on Nature Portfolio policies, see our Editorial Policies and the Editorial Policy Checklist.

Statistics

For all statistical analyses, confirm that the following items are present in the figure legend, table legend, main text, or Methods section.

n/a	Confirmed	
☐	☒	The exact sample size (*n*) for each experimental group/condition, given as a discrete number and unit of measurement
☐	☒	A statement on whether measurements were taken from distinct samples or whether the same sample was measured repeatedly
☐	☒	The statistical test(s) used AND whether they are one- or two-sided<br>*Only common tests should be described solely by name; describe more complex techniques in the Methods section.*
☐	☒	A description of all covariates tested
☐	☒	A description of any assumptions or corrections, such as tests of normality and adjustment for multiple comparisons
☐	☒	A full description of the statistical parameters including central tendency (e.g. means) or other basic estimates (e.g. regression coefficient) AND variation (e.g. standard deviation) or associated estimates of uncertainty (e.g. confidence intervals)
☐	☒	For null hypothesis testing, the test statistic (e.g. *F*, *t*, *r*) with confidence intervals, effect sizes, degrees of freedom and *P* value noted<br>*Give P values as exact values whenever suitable.*
☒	☐	For Bayesian analysis, information on the choice of priors and Markov chain Monte Carlo settings
☒	☐	For hierarchical and complex designs, identification of the appropriate level for tests and full reporting of outcomes
☒	☐	Estimates of effect sizes (e.g. Cohen's *d*, Pearson's *r*), indicating how they were calculated

Our web collection on statistics for biologists contains articles on many of the points above.

Software and code

Policy information about availability of computer code

Data collection	FACSDiva (BD Biosciences, v8.0.1 and v9.0.1), FACSChorus (BD Biosciences, v1.3), ZEN (Zeiss, v3.3)
Data analysis	CellChat (v1.1.3), Imaris (v9), Prism (Graphpad v8), ImageJ (Wayne Rasband, 1.49v), FlowJo (Tree Star Inc., v10.6.2), PhenoGraph (v3.0) clustering algorithm, R (v.4.0.0), CellRanger (v3.0.2), R/Bioconductor package scater (v.1.16.0), Seurat R package (v.4.0.1), clusterProfiler R/ Bioconductor (v.3.15.3)

For manuscripts utilizing custom algorithms or software that are central to the research but not yet described in published literature, software must be made available to editors and reviewers. We strongly encourage code deposition in a community repository (e.g. GitHub). See the Nature Portfolio guidelines for submitting code & software for further information.

Data

Policy information about availability of data

All manuscripts must include a data availability statement. This statement should provide the following information, where applicable:

- Accession codes, unique identifiers, or web links for publicly available datasets
- A description of any restrictions on data availability
- For clinical datasets or third party data, please ensure that the statement adheres to our policy

The scRNA-seq data generated in this study has been deposited in the BioStudies database (www.ebi.ac.uk/biostudies/) and is available under accession code E-MTAB-11715. GRCh38.9 was used as a reference genome to build the indexes. Processed data files can be downloaded from the figshare platform (https://figshare.com) under 10.6084/m9.figshare.21325737 and explored via an interactive browser at https://immbiosg.github.io/FRCdataExplorer/.

Field-specific reporting

Please select the one below that is the best fit for your research. If you are not sure, read the appropriate sections before making your selection.

☒ Life sciences ☐ Behavioural & social sciences ☐ Ecological, evolutionary & environmental sciences

For a reference copy of the document with all sections, see nature.com/documents/nr-reporting-summary-flat.pdf

Life sciences study design

All studies must disclose on these points even when the disclosure is negative.

Sample size	No sample-size calculation was performed. Sample sizes were determined to be adequate based on the reproducibility between independent experiments and patients and adequate cell numbers of each subset in the RNA-seq data to run comparative analyses. Sample sizes for both single cell and in vitro experiments were based on our experience and common practise in the field (Nat Immunol. 2020 Jun; 21(6):649-659).
Data exclusions	No data points were excluded.
Replication	For analysis of the performed scRNA-seq experiments no batch correction needed to be applied for any of the samples. Therefore we can assume careful and good reproducibility. ScRNA-seq data are derived from 12 patients and 9 independent experiments. All attemps at replication were successfull.
Randomization	Randomization and control of covariants was not relevant in the setting of this study as there was no intervention performed.
Blinding	Blinding was not relevant since data analysis was explorative and no intervention was performed.

Reporting for specific materials, systems and methods

We require information from authors about some types of materials, experimental systems and methods used in many studies. Here, indicate whether each material, system or method listed is relevant to your study. If you are not sure if a list item applies to your research, read the appropriate section before selecting a response.

Materials & experimental systems

n/a	Involved in the study	
☐	☒	Antibodies
☒	☐	Eukaryotic cell lines
☒	☐	Palaeontology and archaeology
☒	☐	Animals and other organisms
☐	☒	Human research participants
☒	☐	Clinical data
☒	☐	Dual use research of concern

Methods

n/a	Involved in the study	
☒	☐	ChIP-seq
☐	☒	Flow cytometry
☒	☐	MRI-based neuroimaging

Antibodies

Antibodies used

Anti-human PDPN PE (eBioscience, Clone: NZ-1.3, Cat#: 12-9381-42, Lot#: 4332768)
Anti-human CD45 PeCy7 (eBioscience, Clone: HI30, Cat#: 25-0459-42, Lot#: 2079970)
Anti-human CD31 Biotin (eBioscience, Clone: WM59, Cat#: 13-0319-82, Lot#: 1994108)
Anti-human CD31 PerCP (Biolegend, Clone: WM59, Cat#: 303132, Lot#: B272399)
Anti-human CD235a PeCy7 (Biolegend, Clone: HI264, Cat#: 349112, Lot#: B274230)
Anti-human CD3 FITC (Biolegend, Clone: UCHT1, Cat#: 300440, Lot#: B279209)
Anti-human CD14 PE-Cy7 (Biolegend, Clone: MSE2, Cat#: 301813, Lot#: B231081)
Anti-human CD19 APC/Fire750 (Biolegend, Clone: HIB19, Cat#: 302258, Lot#:B242981)
Anti-human CD45 APC-Cy7 (BD, Clone: 2D1, Cat#: 560178, Lot#: 2079970)
Anti-human CD324 APC (Biolegend, Clone: 67A4, Cat#: 324107, Lot#: B263115)
Anti-human CD11c A647 (Biolegend, Clone 3.9, Cat#: 301613, Lot#: B208071)
Anti-human CD54/ICAM BV421 (Biolegend, Clone: HA58, Cat#: 353132, Lot#: B350347)
Anti-human CD34 FITC (Biolegend, Clone: 581, Cat#: 343504, Lot#: B356958)
Anti-human CD3 (Dako, polyclonal, Cat#: A0452, Lot#: 20061852)
Anti-human PDPN (eBioscience, Clone: NZ-1.3, Cat#: 14-9381-82, Lot#: 2400405)
Anti-human CD20 A488 (eBioscience, Clone: L26, Cat#: 53-0202-80, Lot#: 2210882)
Anti-human/mouse aSMa eFluor660 (eBioscience, Clone: 1A4, Cat#: 50-9760-82, Lot#: 2060395)
Anti-human/mouse aSMa Cy3 (Sigma, Clone:1A4, Cat#: C6198, Lot#: 0000116745)
Anti-human FBLN1 (Atlas Antibodies, Clone: CL0337, Cat#: AMAb906960, Lot#: MAB-03450)
Anti-human PI16 (Novus Biologicals, Cat#: NBP1-92254, Lot#: A104691)
Anti-human CD4 BUV395 (BD, Clone: SK3, Cat#: 563550, Lot#: 2273355)

Anti-human CD8 BUV805 (BD, Clone: SK1, Cat#: 612889, Lot#: 1298376)
Anti-human CD25 BUV563 (BD, Clone: 2A3, Cat#: 612918, Lot#: 1028315)

Anti-rabbit IgG A488 (Jackson Immunoresearch, Cat#: 711-545-152, Lot#: 158217)
Anti-rat IgG A488 (Jackson Immunoresearch, Cat#: 712-545-150, Lot#: 150327)
Anti-mouse IgG A488 (Jackson Immunoresearch, Cat#: 715-545-150, Lot#: 156010)
Anti-rat IgG A594 (Jackson Immunoresearch, Cat#: 712-585-153, Lot#: 152519)
Anti-rat IgG A647 (Jackson Immunoresearch, Cat#: 712-606-150, Lot#: 150018)
Anti-mouse IgG A647 (Jackson Immunoresearch, Cat#: 715-605-150, Lot#: 158693)
Anti-rabbit IgG Cy3 (Jackson Immunoresearch, Cat#: 711-165-152, Lot#: 157936)
Anti-rat IgG Cy3 (Jackson Immunoresearch, Cat#: 712-167-003, Lot#: 144072)
Anti-mouse IgG Cy3 (Jackson Immunoresearch, Cat#: 715-165-150, Lot#: 155993)

Validation

All antibodies came from commercial vendors, and were validated by the manufacturers on their official website. For stainings that used a combination of primary and secondary antibodies, each primary antibody was additionally validated by performing control stains using the secondary antibody alone to ensure a specific signal.

Human research participants

Policy information about studies involving human research participants

Population characteristics

Detailed information is listed in Extended data Table 1 "Characteristics of pediatric and adult patients with obstructive sleep apnea or tonsillitis"

Recruitment

Tonsil samples were collected from adult and pediatric patients suffering from obstructive sleep apnea (OSA) or tonsillitis that underwent routine tonsillectomy at the Kantonsspital St. Gallen. Decision to perform surgery was made after clinical assessment by attending ENT physicians. For the tonsils acquired at UPENN (University of Pennsylvania, Philadelphia, USA) and included in the extended cohort tonsil use was approved by a material transfer agreement between the University of Pennsylvania and Children's Hospital of Philadelphia (ID: 58590/00). These tonsils were received without any protected health information or identifiers, exempt from review by the Children’s Hospital of Philadelphia Institutional Review Board (SOP 407, Section XII “Secondary Use of De-Identified Data or Specimens”).

Ethics oversight

The study protocol has been reviewed and approved by the Ethikkommission Ostschweiz (EKOS), permission numbers 2017-00051 (adult patients) and 2018-01646 (pediatric patients). All study participants provided written informed consent in accordance with the Declaration of Helsinki and the International Conference on Harmonization Guidelines for Good Clinical Practice. All regulations were followed according to the Swiss authorities and according to the clinical protocols.

Note that full information on the approval of the study protocol must also be provided in the manuscript.

Flow Cytometry

Plots

Confirm that:

- [x] The axis labels state the marker and fluorochrome used (e.g. CD4-FITC).
- [x] The axis scales are clearly visible. Include numbers along axes only for bottom left plot of group (a 'group' is an analysis of identical markers).
- [x] All plots are contour plots with outliers or pseudocolor plots.
- [x] A numerical value for number of cells or percentage (with statistics) is provided.

Methodology

Sample preparation

A description of the sample preparation for flow cytometry and FACS sorting is detailed in the methods section.

Instrument

LSR Fortessa BD Biosciences, FACS Melody BD Biosciences, FACSymphony A3 BD Biosciences

Software

FACSDiva (BD Biosciences, v8.0.1 and v9.0.1) was used to collect the data and FlowJo software v10.6.2 (Tree Star Inc.) to analyze the data. FACSChorus (BD Biosciences, v1.3) was used to set up cell sorting, and R v.4.0.0 was used to analyze the transcriptomic data.

Cell population abundance

High purity of the post-sort fraction was confirmed by downstream scRNA-seq analysis.

Gating strategy

For all flow cytometric analysis, cells were first gated on FSC/SSC to exclude cell debris following by FSC-A/FSC-H and SSC-A/SSC-H to exclude doublets. Dead cells were excluded from analysis by gating on viability dye negative staining. Gating strategy for identifying stromal cell populations in this study is exemplifying in the Extended data figures.

- [x] Tick this box to confirm that a figure exemplifying the gating strategy is provided in the Supplementary Information.
